# Recent advances in understanding how compulsivity is related to behavioural addictions over their timecourse

**DOI:** 10.1007/s40429-025-00621-2

**Published:** 2025-02-25

**Authors:** Jeremy E. Solly, Lucy Albertella, Konstantinos Ioannidis, Naomi A. Fineberg, Jon E. Grant, Samuel R. Chamberlain

**Affiliations:** 1 Department of Psychiatry, Faculty of Medicine, University of Southampton, Southampton, UK; 2 Hampshire and Isle of Wight Healthcare NHS Foundation Trust, Southampton, UK; 3 Cambridgeshire and Peterborough NHS Foundation Trust, Cambridge, UK; 4 Department of Psychiatry, University of Cambridge, Cambridge, UK; 5 BrainPark, Turner Institute for Brain and Mental Health, Monash University, Clayton, VIC Australia; 6 School of Life and Medical Sciences, University of Hertfordshire, Hatfield, UK; 7 Hertfordshire Partnership University NHS Trust, Hatfield, UK; 8 Cambridge University School of Clinical Medicine, Addenbrooke’s Hospital, Cambridge, UK; 9 Department of Psychiatry and Behavioral Neuroscience, University of Chicago, Chicago, IL USA

**Keywords:** Compulsivity, Behavioural addiction, Problematic behaviours, Cognitive flexibility, Gambling disorder, Ecological momentary assessment

## Abstract

**Purpose of Review:**

Behavioural addictions involve loss of control over initially rewarding behaviours, which continue despite adverse consequences. Theoretical models suggest that these patterns of behaviour evolve over time, with compulsive and habitual behaviours held to reflect a loss of behavioural control. Compulsivity can be broadly described as a propensity for (or engagement in) repetitive behaviours that are not aligned with overall goals. Here, we consider whether compulsivity is associated with behavioural addictions at different stages of their development, based on self-report and neurocognitive measures.

**Recent Findings:**

This review found that there is initial evidence that compulsive traits might predispose individuals to engage in problematic behaviours, and that self-report and neurocognitive measures of compulsivity are associated with severity of problematic behaviours even in the early stages of behavioural addictions. In the later stages of behavioural addiction, there is strong evidence for an association of gambling disorder with cognitive inflexibility, but less evidence for an association between compulsivity and other types of behavioural addiction.

**Summary:**

Moving forwards, well-powered longitudinal studies, including studies using ecological momentary assessment (EMA), will be important in robustly developing our understanding of how compulsivity is related to behavioural addictions over their timecourse.

## Introduction

Behavioural addictions refer to disorders in which individuals lose control over a behaviour, which continues despite negative consequences and causes distress and/or functional impairment [1]. The behaviour is often rewarding, at least initially; however, the rewarding effects may diminish over time and the behaviour may become more habitual or compensatory [2]. There is growing evidence that behavioural addictions exhibit phenomenological and neurobiological similarities with substance use disorders (SUDs) [3] and this has recently been reflected in diagnostic classification manuals. The 11^th^ revision of the International Classification of Diseases (ICD-11) introduced “disorders due to substance use or addictive behaviours” as a new category, which currently includes gambling and gaming disorders as specified addictive behaviours [1], while the fifth edition of the Diagnostic and Statistical Manual of Mental Disorders (DSM-5) includes gambling disorder in the category “substance-related and addictive disorders” [4]. In addition to gambling and gaming, there is a range of other candidate behavioural addictions, including problematic buying, sexual behaviour (including pornography use) and social networking [2], which can occur in both the online and offline environments [5].

Compulsivity is a construct that is essential for understanding mechanisms or psychological processes by which people exhibit repetitive behaviours even when such behaviours lead to adverse consequences and/or are not in line with their overall goals [6]. The mechanisms underlying compulsive behaviours are debated, but are likely to involve an imbalance between goal-directed (action-outcome) and habit (stimulus-response) learning systems (see [7] and [8] for detailed discussions). For example, evidence from substance addictions and obsessive-compulsive disorder (OCD) supports the hypothesis that compulsive behaviours are associated with decreased goal-directed and increased habit learning [9]. It is important to note that other factors are likely to be involved, as such models do not necessarily fully explain the subjective “loss of control” that often accompanies compulsive behaviours [1, 4, 7, 9]. The drive to engage in such behaviours may involve any, or a combination, of: the development of habits (in which a stimulus and response are paired regardless of the outcome or environmental contingencies and can feel “automatic” [9]); feeling that one must act to avoid harm or reduce anxiety (such as in OCD); or craving to gain a reward [6, 7, 10]. There has been a lack of consensus regarding how compulsivity should be defined [6], in part because it is a complex, multifaceted construct.

Some facets of compulsivity can be assessed using self-report scales. Scales developed to assess obsessive-compulsive symptoms or traits, such as the Padua Inventory [11], are sometimes used as a proxy for compulsivity more generally, although this is potentially problematic since they include factors such as obsessions and compulsive checking or hoarding, which are not relevant to other forms of compulsivity [12]. More recently, scales have been designed to assess compulsivity transdiagnostically and fall into two broad categories. Firstly, the Cambridge-Chicago Compulsivity Trait scale (CHI-T) covers broad aspects of compulsivity not related to specific behaviours across multiple domains including perfectionism (focusing on completeness and high standards), cognitive rigidity (repetitive patterns of thoughts/behaviours) and reward drive (acting based on urges to gain rewards) [13–15]. Secondly, new scales such as the Brief Assessment Tool of Compulsivity Associated Problems (BATCAP) and Granada Assessment for Cross-domain Compulsivity (GRACC) have focused instead on measuring compulsive aspects of specific behaviours, such as time lost, loss of control, and urges [16, 17]. For the purposes of this review, we use the term “general compulsivity” to relate to compulsivity measures *unrelated* to a specified behaviour (i.e., are deliberately transdiagnostic in nature) and “behaviour-specific compulsivity” to relate to compulsivity measures *related* to a specific behaviour. General compulsivity and behaviour-specific compulsivity would be expected to correlate with each other to some degree, based on the premise that a transdiagnostic measure of compulsivity would be expected to predispose to manifestations of different related compulsive behaviours. Indeed initial work has found this – for example, the CHI-T correlates positively with BATCAP scores for alcohol use, gambling and binge eating [18].

Other facets of compulsivity can be assessed using neurocognitive tasks. Individual tasks often examine a range of executive functions, such as the abilities to update working memory, inhibit prepotent responses and shift mental sets [19]. For example, the Wisconsin Card Sort Test (WCST) [20] is classically viewed as a test of set-shifting ability (i.e., cognitive flexibility), but also involves ability to learn rules, categorise stimuli and inhibit learned responses following a change in feedback [21]. Facets of compulsivity amenable to examination with measures from neurocognitive tasks include a tendency to follow rigid strategies, deficiencies in attentional set-shifting, persistence of habits, perseveration of unrewarded responses (e.g., in reversal learning paradigms), and impaired motor inhibition or ability to stop a prepotent response [22]. It is important to note that not all of these psychological mechanisms are specific to compulsivity: while set-shifting and reversal learning are classically considered facets of compulsivity, the ability to inhibit prepotent responses overlaps with other behavioural constructs, such as impulsivity [22]. Thus, compulsivity is associated with executive dysfunction, and therefore loss of control over behaviour, but is not the only mechanism/construct related to such loss of control.

Most operationalisations of neurocognitive tasks relate to general (as opposed to behaviour-specific) compulsivity, as they use generic test stimuli differing in dimensions such as colour, number and shape. Classic tasks such as the WCST and intra-extradimensional set-shift task (IED) [23] require participants to shift their attention between different dimensions of test stimuli, while the the Trail-Making Test part B (TMT-B) [24] requires participants to alternate their focus between letters and numbers. Measures from these tasks, such as the numbers of errors (WCST and IED) or the time taken to complete the task (TMT-B) therefore can relate to an ability to flexibly shift one’s attention [25]. The probabilistic reversal learning task (PRL) [26] requires participants to adapt their behaviour to rule changes, and so the number of perseverative errors after a rule change relates to a participant’s ability to flexibly update their behaviour [25]. Other approaches have also been developed to assess additional facets of compulsivity. For example, the two-step task assesses the extent to which participants act using a model-based “goal-orientated” strategy or a model-free “habitual” strategy when exploring an environment to gain rewards, with a more model-free strategy suggestive of higher compulsivity [27]. The value-modulated attentional capture task (VMAC) tests the extent to which participants are distracted from their task by a stimulus denoting whether the trial will have a low or high reward, which reflects a tendency toward cue-triggered maladaptive (compulsive) behaviour [16]. Tests measuring the inhibition of prepotent responses are widely considered to capture impulsivity [28]. However, in some settings, failure of inhibition on relevant tasks can be considered to relate to compulsivity (e.g., failure to suppress an established habitual response on the task) [22].

## Theoretical models of the role of compulsivity in behavioural addictions

The transition of behaviour which is initially driven by positive reinforcement but then becomes habitual and compulsive is an essential component of addiction [7]. The same notion is also described in a well-known model from the substance addiction literature, here termed the “addiction stages” model, in which engagement with the substance/problematic behaviour is initially rewarding, but escalated use and dependence involve a shift from goal-directed towards habitual responses to addiction-related cues [29]. The Interaction of Person-Affect-Cognition-Execution (I-PACE) model, which was developed specifically for behavioural addictions, elaborates on the idea that addictions develop in stages [2]. In fact, the new I-PACE model was specifically updated to include this distinction and stresses the importance of loss of inhibitory control in the addiction development process [2, 30]. It suggests that a person’s characteristics are predisposing factors and that, when these interact with the environment, affective and cognitive responses to behaviour-specific stimuli result in a decision to engage in the behaviour, which may lead to gratification or relief of negative affect. In the early stages of the addiction, a lack of general inhibitory control could mediate repetitive engagement in the problematic behaviour while, in the later stages, the transition to habitual behaviour is mediated in part by a lack of general inhibitory control, but also by cue-reactivity and craving in response to behaviour-specific triggers leading to stimulus-specific impairment in inhibitory control [2]. Recently, a simpler model (based on the I-PACE) has posited a more explicit role for compulsivity in driving behavioural addictions [31]. In this model, Brand (2022) suggests that in the later stages of addiction a “must do” (compulsive) pathway involving the dorsal striatum overrides a “stop now” inhibitory control process involving the dorsolateral prefrontal cortex, leading to an individual engaging in excessive, uninhibited habitual or compulsive behaviours despite negative consequences [31].

These models suggest that different facets of compulsivity could play roles in different stages of the development of an addiction. Firstly, general compulsivity can be conceptualised as a trait, describing a tendency toward repetitive actions despite adverse consequences, that can be measured in the general population [32]. This aspect of compulsivity could therefore act as a predisposing factor for the development of a behavioural addiction. When considering the early stages of an addiction, the I-PACE model posits a role for a lack of general inhibitory control [2], which is related to facets of compulsivity such as behavioural and attentional inflexibility, urge-driven engagement in behaviour and a tendency to develop habits. In the later stages of an addiction, both the addiction stages and the I-PACE models suggest an important role for habitual responses to addiction-related cues, in which general compulsivity plays a role but behaviour-specific compulsivity may become relatively more prominent.

In this review, we consider recent evidence for the role of compulsivity in behavioural addictions. Informed by the addiction stages and I-PACE models, we consider whether different facets of compulsivity are relevant at different stages of the addiction process and discuss the next steps for improving our understanding of the relationship between compulsivity and behavioural addictions.

## Compulsivity as a predisposing factor for behavioural addictions

The “person” component of the I-PACE model suggests that general predisposing variables, such as genetics, temperament and pre-existing psychopathology, interact with specific motivations and triggers to drive repetitive engagement in a problematic behaviour [2]. A tendency towards compulsive responding could act as one of these predisposing factors. For example, an individual who is cognitively inflexible might be less able to change their behaviour once they realise that an initially rewarding behaviour has started to become problematic [33].

Firstly, genetic approaches are an important avenue for considering compulsivity as a predisposing factor in the development of behavioural addictions. Some studies have approached this by considering relationships of behavioural addictions with other disorders characterised by compulsivity, such as OCD and substance use disorders. A twin study demonstrated that gambling disorder and OCD symptoms overlapped and that common genetic variance contributed to this association [34]. A study of young people who gambled found that a family history of substance use disorder was associated with losing more money through gambling, more symptoms of gambling disorder, and more IED errors [35]. These findings support the existence of a heritable predisposition to compulsive behaviors and inflexible thinking patterns. Indeed, higher CHI-T scores are associated with family history of both addictions and obsessive-compulsive and related disorders [18], suggesting that trait compulsivity could be part of this picture. The Psychological and Genetic Factors of Addictions study, which considered 32 single nucleotide polymorphisms, identified that the *FOXN3* gene is associated with alcohol use, problematic internet use, and gaming disorder [36].

While these findings can be seen as exploratory at this stage, important next steps will be to replicate these results and identify genetic determinants for behavioural addictions via twin studies or studies of family histories as well as genetic polymorphism studies, which can identify variants transdiagnostically associated with a range of behavioural addictions, as well as with trait compulsivity. In the absence of well-powered genome wide association studies focusing on behavioural addictions, a fruitful next step may be to assess whether polygenic risk scores for other compulsive spectrum or addictive disorders (such as OCD [37] and alcohol use [38]) are associated with behavioural addictions. This could point towards shared genetic aetiology, as has been used to demonstrate genetic associations among substance use disorders [38, 39]. Challenges for the future will include understanding whether such genetic associations relate specifically to behavioural addictions, or to factors such as general compulsivity, which are associated with a range of mental disorders. Another important future step will be to integrate these potential genetic insights into biopsychosocial models for different behavioural addictions [40].

A second important approach for identifying predisposing factors is the use of longitudinal studies following individuals over time to examine whether particular traits or exposures are associated with the development of a behavioural addiction. To our knowledge, little longitudinal work such as this has been completed to date. A study following young people in the UK over a period of 4–6 years found that obsessive-compulsive symptoms at baseline were associated with behaviours such as repeating actions over and over again, ordering, planning, and checking, but not with gambling at follow-up [41]. In a three-year longitudinal study of young adults who engaged in gambling, higher compulsivity at baseline – as measured both by the Padua Inventory and IED task – was associated with persistence of gambling disorder symptoms at follow-up [42]. To robustly tease out predisposing factors for behavioural addictions, significantly longer term studies with large sample sizes will be required, ideally starting in childhood before engagement in potentially problematic behaviours begins and including both questionnaires and neurocognitive tasks to assess different facets of compulsivity.

## Compulsivity in the early stages of behavioural addiction

The I-PACE model suggests that a lack of general inhibitory control, which implicates compulsivity, is important in moderating the relationship between affective responses/environmental cues and repeated engagement with a behaviour in the early stages of an addiction. The lack of well-powered longitudinal studies capturing the emergence of behavioural addictions means there is little direct evidence about the potential role of compulsivity in these early stages. Behavioural addictions as defined in diagnostic manuals do not exist in isolation but represent the severe end of a spectrum of engagement in potentially problematic behaviours. For example, engagement in gambling can be considered along a spectrum encompassing ‘recreational’ gambling, problematic (or hazardous) gambling, and gambling disorder [1]. For an individual to develop gambling disorder, they must have access to gambling, begin engaging in it, increase their participation, and continue or escalate gambling despite adverse effects [1]. This means that a proportion of those exhibiting hazardous engagement in a potentially addictive behaviour at any given time are in the early stages of a behavioural addiction, although it is important to note that problematic engagement in a behaviour can persist or improve over time without the development of a diagnosable behavioural addiction [42]. Despite these caveats, in the absence of more robust longitudinal evidence, for the purposes of this review we will consider two types of studies as potentially useful in understanding the early stages of behavioural addictions: (1) cross-sectional studies considering dimensional relationships between compulsivity and problematic behaviours in community samples, and (2) cross-sectional studies including groups with problematic engagement in a behaviour not reaching the threshold for a (clinical) addiction diagnosis.

When considering a potential dimensional relationship between general compulsivity and behavioural addiction symptoms in community samples, recent evidence supports the hypothesis that compulsivity is associated with higher levels of engagement in problematic behaviours. CHI-T scores positively correlate with symptoms of disordered gambling and pornography use, as well as other potentially problematic behaviours such as internet use and disordered eating [18, 43–45]. When considering reward-related attentional capture using the VMAC task in an online sample, greater distraction by higher reward indicators was positively correlated with behavioural addiction symptoms [18], suggesting that a tendency towards repetitive maladaptive cue-triggered behaviours could be a component in the early stages of an addiction. A strength of community samples is their ability to demonstrate the importance of considering other factors, such as the environment, when exploring the role of compulsivity in the development of behavioural addictions. For example, CHI-T score was associated with problematic gambling pre-COVID, but overall levels of problematic gambling in the population decreased during lockdown, and increased lockdown problematic gambling was more strongly associated with younger age and higher levels of psychological distress than with compulsivity [43]. Thus, future research aiming to understand the role of compulsivity in the early stages of behavioural addictions should consider the environment as a key factor, for example by including populations exposed to different regulatory environments or different levels of internet use.

Moving to studies considering sub-clinical groups, there is emerging evidence for compulsivity as an important factor in such subthreshold symptoms. In a study of young adults who engaged in gambling, those with intermediate symptoms (which did not meet criteria for gambling disorder) had higher scores on the Padua Inventory and made more IED errors than those who reported low symptoms at baseline, suggesting that compulsivity, including cognitive inflexibility, might be an important factor in a transition from recreational to disordered gambling [42]. Another recent study considered the cross-sectional relationship between self-report compulsivity, measured using the obsessive-compulsive subscale of the Symptom Check List (SCL-90), and symptom severity for a range of problematic behaviours [46]. This identified that those endorsing symptoms of gambling disorder or exercise dependence without meeting the full suggested diagnostic criteria had higher compulsivity scores than those who were asymptomatic. Although this study also considered buying, gaming and internet use, these behaviours were fractionated into only two severity levels (non-problematic vs. problematic, or non-pathological vs. pathological) [46]. More studies specifically considering the factors associated with problematic subthreshold symptoms of behavioural addictions are needed and will be useful both for improving our understanding of the development of behavioural addictions, as well as for targeting harm reduction interventions to those at risk of developing disorders.

## Compulsivity in the later stages of behavioural addictions

In the later stages of an addiction, the addiction stages model suggests that behaviour becomes habitual and compulsive in response to addiction-related cues, while the I-PACE model postulates roles for deficits in both general inhibitory control and behaviour-specific inhibitory control, in which there is a reduction in stimulus-specific inhibitory control associated with affective processes [2]. In this section, we first consider the current evidence for an association of behaviour-specific compulsivity with the later stages of behavioural addiction, before considering associations of facets of general compulsivity with these later stages. Here, we view the “later” stages of development of a behavioural addiction as involving symptoms that meet the criteria for a diagnosis.

### Behaviour-specific compulsivity

Behaviour-specific compulsivity, in which specific cues related to a problematic behaviour interact with cognitive control, has not been overtly considered by most methods used for measuring compulsivity to date. To our knowledge, there are no published studies which use behaviour-specific stimuli in a task assessing compulsivity in a sample with a behavioural addiction meeting the threshold for diagnosis. Instead of incorporating behaviour-specific stimuli into the task itself, one study in compulsive sexual behaviour paired a behaviour-specific exposure with the WCST, finding no case-control difference at baseline, but worse WCST performance in those with compulsive sexual behaviour after an erotic video exposure [47]. In future, studies using behaviour-specific neurocognitive tasks will be required to assess whether, and how, cue-reactivity affects facets of neurocognitively measured compulsivity such as attentional inflexibility. However there are many challenges in attempting to develop such methodologies. The content of addictive triggers/cues varies considerably from person to person, across many behavioural addictions, and can also fluctuate markedly over time in a given individual too. Thus, it can be extremely challenging to develop and validate paradigms capabable of capturing these processes in a reliable, reproducible way across a range of participants, disorders, and settings.

Self-report scales designed to capture symptoms of behavioural addictions do not focus exclusively on compulsive facets of the symptomatology; for example, the Gambling Symptom Assessment Scale (G-SAS) includes items related to excitement/pleasure when winning bets, and functional impairment [48]. The recent development of self-report measures aimed at capturing compulsive symptoms of specific behavioural addictions, such as the BATCAP and GRACC [16, 17], lays the groundwork for future studies considering the relative balance of compulsive symptoms at different stages of behavioural addictions.

### General compulsivity

In contrast to the lack of evidence regarding behaviour-specific compulsivity in samples meeting criteria for behavioural addictions, there is more evidence for an association of general compulsivity with this “later” stage of behavioural addiction.

Neurocognitive tasks assessing a general ability to shift attention between stimuli have been widely used in case-control studies, particularly in gambling disorder [25, 49]. Meta-analytic evidence demonstrates that gambling disorder is associated with impaired performance in the WCST, IED and TMT-B [25]. More recent studies continue to support the general association of gambling disorder with attentional set-shifting deficits. Two studies of treatment-seeking patients with gambling disorder based at the same centre demonstrated WCST performance deficits [50, 51], although there was no case-control difference in TMT-B performance [51]. In a study of young adults from the general community who gambled, the presence of established gambling disorder was associated with a deficit in IED performance, including in the crucial extradimensional shift stage in which participants are required to shift their attention to a new stimulus dimension [52]. As all participants in this study endorsed gambling at least occasionally, this finding supports the conclusion that cognitive inflexibility is – at least to some degree – associated with established gambling disorder, than with gambling *per se*. However, this was a non-treatment seeking sample and the effect sizes reported were small. In addition, those deficits also mapped (even more strongly) to other clinical entities (e.g., post-traumatic stress disorder or generalised anxiety disorder), which makes them less specific as predictive biomarkers of gambling.

Turning to other types of behavioural addiction, gaming disorder has recently been associated with impaired performance in both the WCST and IED [53, 54], although previous work found an IED deficit in gambling but not gaming [55], and to our knowledge there is no relevant meta-analysis. A recent review of neurocognition in compulsive buying found no evidence for impaired performance in the WCST or TMT-B [56], and a study in patients with binge-spectrum eating disorders comparing those with and without compulsive buying found no difference in WCST performance [57]. Recent studies in compulsive sexual behaviour and problematic social networking have not shown case-control differences in WCST performance under neutral experimental conditions [47, 58].

Moving to ability to adapt behaviour to rule changes in tasks involving degraded probabilistic feedback, as in the PRL task, meta-analysis has found mixed results for gambling disorder-associated performance deficits [25]. These meta-analysed studies reported overall task outcomes such as number of correct choices [25], while more recent studies have used alternative analysis frameworks. When trial-by-trial performance was considered, gambling disorder participants learned more slowly after a rule change and made decisions more randomly, with stronger tendencies both to switch choices when there was a lack of negative feedback, as well as to perseverate despite accumulating negative feedback [59]. Studies applying a computational approach to probabilistic learning in gambling disorder have demonstrated lower stimulus “stickiness”, reflecting a tendency to switch choices regardless of the outcome [60], as well as increased learning from better-than-expected and decreased learning from worse-than-expected outcomes [61]. Together, these findings suggest that PRL performance deficits in gambling disorder likely have several components, including impaired reversal learning, a tendency to give too much weight to positive outcomes, and inflexibly following a “switch” strategy while disregarding feedback.

In addition to applying novel analysis strategies to classical neurocognitive tasks, new methods of assessing different facets of compulsivity have recently contributed to our understanding of its association with gambling disorder. For example, the two-step task, which assesses whether participants tend to act using a model-based “goal-orientated” strategy or a model-free “habitual” strategy [27], was recently used to show that model-based decision making was reduced after negative outcomes in gambling disorder, indicating an over-reliance on habit specifically when not rewarded [62]. Additionally, when considering self-report scales, gambling disorder was associated with higher scores on the CHI-T in university students [63], supporting the conclusion that both neurocognitive and trait-based facets of compulsivity are associated with established gambling disorder.

It is important to note that, aside from neurocognitive measures of attentional flexibility, there is a paucity of recent research exploring associations of behavioural addictions other than gambling disorder with facets of general compulsivity. This may reflect the fact that behavioural addiction models until recently lacked conceptualizations which included compulsivity in their main components [64]. Future studies should consider both neurocognitive and self-report measures, with a view to clarifying whether different behavioural addictions are associated with similar or different facets of compulsivity.

## “Hot” dynamic compulsivity

When considering compulsivity across the timecourse of behavioural addictions, it is essential to consider how this might fluctuate over time. Despite the recognition that cognitive-affective interactions can promote compulsive behaviours in real time, the majority of the current knowledge base on cognitive risk factors has come from ‘static’ methods that use ‘single-shot’ assessments, which assume that cognition is largely stable (at least in the short-term) and can be reliably measured at a single point in time to reflect an individual’s current level of cognition for a given domain (e.g., cognitive control) [65, 66]. However, this is not always the case, and the over-reliance on single-shot approaches may have contributed to the field’s inconsistent/mixed findings and the relative lack of high-precision insights to date. Even if one were solely interested in an individual’s average-level cognition (and not fluctuations *per se*), a single-shot assessment of cognition would be unlikely to reflect that person’s current average [66]. This might especially be the case in the context of compulsivity, in which related traits (such as high trait neuroticism) are associated with greater negative affect variability and in turn greater cognitive variability [67]. This can even occur at the trial level, with task features (such as errors) capable of triggering changes in affective states and thereby performance deficits and inconsistencies [68, 69].

Compulsive behaviours typically occur in the context of strong emotional states such as stress, highlighted by phenomena such as stress-induced relapse and stress-induced urges and symptoms [70–74]. These phenomena have been interpreted as arising from the disruptive effects of strong negative affect on cognitive control [75–79], which in turn can bias ongoing behaviour towards compulsivity (for a review, see [70]). For instance, stress can bias attention toward compulsivity-related stimuli (e.g., websites, slot machines, etc), even when they do not align with an individual’s goals. Stress can also change an individual’s goals toward stress-reduction [78]. Stress aside, other mental states (such as mental fatigue) can also induce temporary changes in cognition such as disruptions in response inhibition and in turn may temporarily reduce control over compulsive behaviours [70, 80].

The issue of stress-induced cognitive variability underlines the need for methods that capture these dynamic cognitive-affective patterns as they play out in real time to gain more precise mechanistic insights. Toward this aim, ecological momentary assessments (EMA) of cognition, affect, and compulsive behaviours over days can reveal new insights into the dynamic mechanisms of compulsivity as well as offer just-in-time interventions to target real-time risk. Indeed, while rare, cognitive EMA studies in this space are showing the potential of these methods, such as a recent study showing that momentary response disinhibition drives snacking behaviours in real time [81], and another study showing that momentary fluctuations in attentional disinhibition predicted momentary fluctuations in behaviour and emotion regulation [82].

## Conclusions

**Fig. 1:**
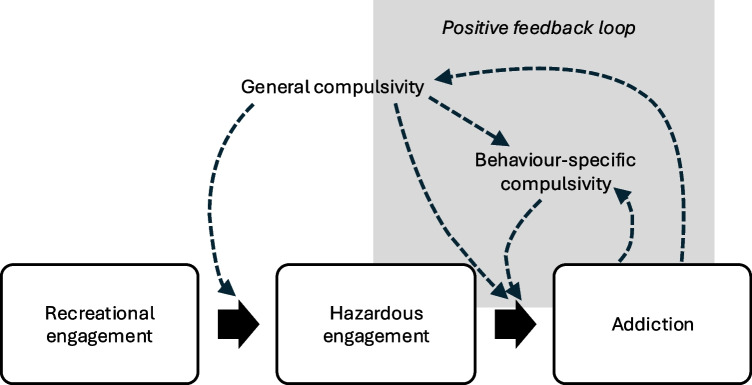
Potentially problematic behaviours can be considered along a spectrum including recreational engagement, hazardous engagement, and engagement meeting criteria for an addiction diagnosis. In the early stages of the development of a behavioural addiction, general compulsivity may drive repetitive engagement. In the later stages, both general and behaviour-specific compulsivity may contribute to the severity and/or chronicity of addiction symptoms. The dashed arrows represent hypothetical directional relationships which require testing in future work. Of particular importance may be a potential positive feedback loop (grey box), in which compulsivity predisposes to development of an addiction, and repetitive engagement in the addictive behaviour then strengthens brain mechanisms driving compulsive behaviours [31]

Based on the premise that behavioural addictions develop in stages (Fig. 1), and informed by the addiction stages and I-PACE models [2, 29], this review has aimed to synthesise recent research to explore the associations of compulsivity with behavioural addictions across their timecourse. When considering compulsivity as a predisposing factor in the development of behavioural addictions, there is some evidence of shared genetic variance between behavioural addictions and other compulsive disorders, such as OCD. In the early stages of behavioural addictions, there is also some evidence for an association with trait self-report compulsivity, as well as with reward-related attentional capture and attentional set-shifting deficits. Finally, in the later stages of behavioural addictions, gambling disorder is associated with deficits in neurocognitive task performance related to a variety of facets of compulsivity, such as attentional set-shifting, adapting to rule changes, and an over-reliance on habit.

While there is robust meta-analytic evidence for cognitive inflexibility in established gambling disorder [25], these findings are largely cross-sectional and so cannot clarify our understanding of the direction of this relationship, albeit there is initial evidence that baseline inflexibility is associated, to some degree, with persistence of gambling disorder symptoms over time in young adults who gamble [42]. Taking into account the multifaceted and fluctuating nature of compulsivity over time, it will be important in future work to investigate whether different facets of compulsivity represent vulnerability factors and/or evolve during the course of behavioural addictions (Fig. 1) [31]. Understanding such directionality is of great clinical relevance, as targeting general *vs.* behaviour-specific compulsivity may require different interventions. Clarifying these relationships requires large-scale longitudinal studies with validated measures. An ongoing example of such a study is BootStRaP (www.internetandme.eu), which focuses specifically on understanding problematic internet usage in young people and will collect properly validated self-report and neurocognitive measures related to compulsivity over time. Unfortunately, many of the ‘well-known’ large scale longitudinal population studies have not included validated self-report trans-diagnostic measure(s) of compulsivity to date. In addition to conventional case-control studies and longitudinal cohort work using standard self-report and neurocognitive metrics of compulsivity, EMA approaches could play a vital role in helping to understand how symptoms fluctuate dynamically, including in relation to compulsivity, in turn potentially leading to novel treatment approaches in future.

## Key References

- Brand M, Wegmann E, Stark R, Müller A, Wölfling K, Robbins TW, et al. The interaction of person-affect-cognition-execution (I-PACE) model for addictive behaviors: update, generalization to addictive behaviors beyond internet-use disorders, and specification of the process character of addictive behaviors. Neurosci Biobehav Rev. 2019;104:1–10.
  - º The updated I-PACE model, which presents a detailed theoretical basis for studies of behavioural addiction.
- Tiego J, Trender W, Hellyer PJ, Grant JE, Hampshire A, Chamberlain SR. Measuring Compulsivity as a Self-Reported Multidimensional Transdiagnostic Construct: Large-Scale (N = 182,000) Validation of the Cambridge–Chicago Compulsivity Trait Scale. Assessment. 2023;30:2433–48.
  - º A large-scale validation of the CHI-T, which allows transdiagnostic assessment of broad aspects of compulsivity not related to specific behaviours.
- Albertella L, Le Pelley ME, Chamberlain SR, Westbrook F, Fontenelle LF, Segrave R, et al. Reward-related attentional capture is associated with severity of addictive and obsessive–compulsive behaviors. Psychol Addict Behav. 2019;33:495–502.
  - º This study demonstrates the relevance of the VMAC task to compulsive behaviours.
- van Timmeren T, Daams JG, van Holst RJ, Goudriaan AE. Compulsivity-related neurocognitive performance deficits in gambling disorder: A systematic review and meta-analysis. Neurosci Biobehav Rev. 2018;84:204–17.
  - º A systematic review and meta-analysis of 29 studies, which indicates that gambling disorder is associated with compulsivity-related neurocognitive deficits.
- Demetrovics Z, van den Brink W, Paksi B, Horváth Z, Maraz A. Relating Compulsivity and Impulsivity With Severity of Behavioral Addictions: A Dynamic Interpretation of Large-Scale Cross-Sectional Findings. Front Psychiatry. 2022;13:831992.
  - º A transdiagnostic study which examines the associations of compulsivity and impulsivity with symptom severity in a range of behavioural addictions and candidate behavioural addictions.
- Munoz E, Stawski RS, Sliwinski MJ, Smyth JM, Macdonald SWS. The Ups and Downs of Cognitive Function: Neuroticism and Negative Affect Drive Performance Inconsistency. J Gerontol B Psychol Sci Soc Sci. 2020;75:263–73.
  - º A recent study demonstrating that trial-to-trial inconsistency in neurocognitive tasks is associated with neuroticism and negative affect.

## Data Availability

No datasets were generated or analysed during the current study.
